# Gene Expression Signature in Adipose Tissue of Acromegaly Patients

**DOI:** 10.1371/journal.pone.0129359

**Published:** 2015-06-18

**Authors:** Irit Hochberg, Quynh T. Tran, Ariel L. Barkan, Alan R. Saltiel, William F. Chandler, Dave Bridges

**Affiliations:** 1 Institute of Endocrinology, Diabetes and Metabolism, Rambam Health Care Campus, Haifa, Israel; 2 Life Sciences Institute, University of Michigan, Ann Arbor, MI, United States of America; 3 Department of Preventive Medicine, University of Tennessee Health Science Center, Memphis, TN, United States of America; 4 Department of Internal Medicine, University of Michigan, Ann Arbor, MI, United States of America; 5 Department of Neurosurgery, University of Tennessee Health Science Center, Memphis, TN, United States of America; 6 Department of Physiology, University of Tennessee Health Science Center, Memphis, TN, United States of America; 7 Children's Foundation Research Institute, Le Bonheur Children's Hospital, Memphis, TN, United States of America; University of Alabama at Birmingham, UNITED STATES

## Abstract

To study the effect of chronic excess growth hormone on adipose tissue, we performed RNA sequencing in adipose tissue biopsies from patients with acromegaly (n = 7) or non-functioning pituitary adenomas (n = 11). The patients underwent clinical and metabolic profiling including assessment of HOMA-IR. Explants of adipose tissue were assayed *ex vivo* for lipolysis and ceramide levels. Patients with acromegaly had higher glucose, higher insulin levels and higher HOMA-IR score. We observed several previously reported transcriptional changes (*IGF1*, *IGFBP3*, *CISH*, *SOCS2)* that are known to be induced by GH/IGF-1 in liver but are also induced in adipose tissue. We also identified several novel transcriptional changes, some of which may be important for GH/IGF responses (*PTPN3* and *PTPN4*) and the effects of acromegaly on growth and proliferation. Several differentially expressed transcripts may be important in GH/IGF-1-induced metabolic changes. Specifically, induction of *LPL*, *ABHD5*, and *NRIP1* can contribute to enhanced lipolysis and may explain the elevated adipose tissue lipolysis in acromegalic patients. Higher expression of *TCF7L2* and the fatty acid desaturases *FADS1*, *FADS2* and *SCD* could contribute to insulin resistance. Ceramides were not different between the two groups. In summary, we have identified the acromegaly gene expression signature in human adipose tissue. The significance of altered expression of specific transcripts will enhance our understanding of the metabolic and proliferative changes associated with acromegaly.

## Introduction

Acromegaly, i.e. excessive growth hormone (GH) production secondary to a pituitary adenoma, is a rare condition with an annual incidence of 3 patients per million [1]. The excess GH has important metabolic effects; the two most significant effects of GH on metabolism in adipose tissue are insulin resistance and lipolysis [2]. Insulin resistance, presenting as diabetes or impaired glucose tolerance, is found in most acromegalic patients [3], and contributes to the enhanced morbidity [4]. Growth hormone induces the expression and secretion of IGF-1, so phenotypes associated with acromegaly may be due to either GH signaling, IGF-1 signaling or a combination of both [5,6].

There are few studies addressing the effect of GH specifically on the subcutanous adipose tissue. Induction of STAT5 tyrosine phosphorylation and IGF1 mRNA expression has been detected in human subcutaneous adipose tissue biopsies taken after acute GH administration [7]. Subcutaneous adipocytes extracted from acromegalic patients are insulin resistant *ex vivo*, and after a glucose tolerance test there was 50% less insulin binding to its receptor and markedly decreased insulin-related anti-lipolytic activity [8]. *In vivo* measurement in humans detected GH-induced lipolysis in subcutanous adipose tissue [9]. Pharmacologic inhibition of lipolysis reduced GH-induced insulin resistance, suggesting that some of this resistance is dependent on higher abundance of free fatty acids [10]. Microarray of gene expression has been published for subcutaneous adipose tissue biopsies before and after one year of GH treatment in GH deficient patients [11].

To study the effects of chronic excess GH, we used unbiased RNA sequencing in adipose tissue from acromegaly patients and controls. We found a distinctive pattern of changes in many transcripts that are highly associated with acromegaly. Many of these alterations may contribute to the metabolic effect of GH and reveal novel mechanisms of GH-induced insulin resistance and lipolysis in adipose tissue.

## Materials and Methods

### Patient Recruitment

The study was approved by the institutional review board of the University of Michigan Medical System. Written informed consent was obtained from all patients. Patients were recruited consecutively from a cohort undergoing transsphenoidal adenomectomy at the University of Michigan Medical Center for acromegaly or non-functioning pituitary adenoma over a 12 month period. All but one patient were newly diagnosed, none had previous surgery and only one previously diagnosed patient was treated with a somatostatin analog but IGF1 was still high without remission. None of the patients were on insulin, but one patient from each group was treated with metformin. Two patients with non-secreting adenomas were treated with beta blockers. Exclusion criteria were age <18 years old, current hormone treatment including glucocorticoids, malignancy, inflammatory disease, diabetes type 1 and established pituitary hormone deficiencies. For each patient, a data sheet was completed including, age, sex, anthropometric measurements, diagnosis of hypertension, diabetes, results of blood tests and medications. Fasting blood samples were assayed for glucose (Siemens Advia 1800) and insulin (Life Technologies) as instructed by the manufacturers.

### Subcutaneous Fat Biopsy

During the course of pituitary surgery a routine subcutaneous fat graft is utilized to seal the surgical field upon completion of the procedure. A total of 500 mg of this fat graft was used for the study. ~200 mg were utilized for ex vivo lipolysis assay, ~300 mg was snap frozen in liquid nitrogen and stored at -80 degrees for RNA preparation and ceramide analysis.

### *Ex vivo* Lipolysis

Twenty five mg pieces of adipose tissue were pre-incubated for 15 minutes in KRBH buffer (sigma) at 37°C and then incubated for 1 hour at 37°C in 300 μl KRBH in the presence or absence of isoproterenol 30nM in duplicate. Glycerol was assayed in supernatants using a glycerol assay kit (sigma) as instructed by manufacturer.

### Ceramide Determinations

Ceramide analysis of tissue samples was performed by liquid chromatography-triple quadrupole mass spectrometry (LC-QQQ) according to a modified version of the protocol reported in [12]. Briefly, frozen tissue samples were pulverized under liquid nitrogen, then 20 mg portions were extracted using 1.6 mL of a 2:1:0.8 mixture of chloroform:methanol:water containing internal standards (50 ng each of C17 and C25 ceramide and C12 glucosylceramide per sample) [13]. The organic layer of the extract was dried under nitrogen gas and reconstituted in 100 uL of 60:40 acetonitrile: isopropanol. The re-constituted extract was analyzed by electrospray ionization LC-MS/MS on an Agilent (Santa Clara, CA) 6410 triple quadrupole instrument operating in positive ion multiple reaction monitoring mode. The LC column used was a Waters (Milford, MA) Xbridge C18 2.5 μ, 50 mm x 2.1 mm i.d. Mobile phase A was 5mM ammonium acetate, adjusted to pH 9.9 with ammonium hydroxide; mobile phase B was 60:40 acetonitrile:isopropanol. The gradient consisted of a linear ramp from 50 to 100%B over 5 minutes, a 20 minute hold at 100%B, and re-equilibration at 50%B for 10 minutes. Injection volume was 25 μL. Ceramides and glucosylceramides were identified by retention time and by MS/MS fragmentation parameters, and were quantitated by peak area relative to the closest-matching internal standard using Agilent MassHunter Quantitative Analysis software.

### Transcriptomic Analysis

Total RNA was extracted from adipose tissue using the RNEasy kit (Qiagen) and its quality was verified using the Agilent 2100 Bioanalyzer (Agilent Technologies). At the University of Michigan DNA Sequencing Core, cDNA libraries from polyA mRNA were prepared using TruSeq cDNA synthesis kit and sequenced using a HiSeq 2000 (Illumina). Samples were run on 2 lanes of a HiSeq 2000 (Illumina) generating 8 612 682 to 16 469 501 single-ended 50 bp reads per sample. These were aligned to the human genome (Enembl GRCh37.74, Genbank Assembly ID GCA_000001405.14) using TopHat version 2.0.10 [14], Bowtie 2 version 2.1.0 [15] and Samtools version 0.1.18. Reads were mapped to known genes using HTseq [16]. Gene expression was analyzed using DESeq2 version 1.2.10 [17]. To account for potential age-dependent changes in the subjects, we separated the patients into two groups, based on the median value, under 60 years of age versus 60 and above as has been previously reported for acromegaly studies [18]. We provide here both the non-age adjusted (S1 Table) and age-adjusted gene expression changes (S2 Table).

We then added this age group as a covariate along with the disease state. We tested for interactions between the age group and the disease state for each gene and did not identify any interaction term after adjusting for multiple observations (q<0.05). All fold changes provided in this manuscript are age-adjusted fold change values calculated from this regression.

We used Gene Set Enrichment Analysis (GSEA v2.0.13 [19,20]) to determine whether our rank-ordered gene list for the comparison of acromegaly vs control patients is enriched in genes from gene ontology, KEGG, transcription factor or microRNA target gene sets. The gene list was ranked based on the shrunken log based 2 fold change and the statistical significance of the enrichment score was determined by performing 1000 phenotype permutations and setting the enrichment statistics to classic. Other settings for GSEA pre-ranked were left by the software default.

These subjects for whom RNAseq was performed corresponded to the patients described in Table 1, with the exception of subjects 29 and 31 (both acromegaly patients), which had clinical data but no RNAseq data. These data are available through the Gene Expression Omnibus (GSE57803).

**Table 1:** Clinical characteristics.

	Control (n = 11)		Acromegaly (n = 9)		p
Age (years)	63.4	+/- 2.7	48.3	+/- 4.9	0.011
Height (cm)	170.0	+/- 2.4	180.1	+/- 4.0	0.036
Weight (kg)	89.4	+/- 6.7	103.9	+/- 9.3	0.21
BMI (kg/m^2^)	30.7	+/- 1.8	31.7	+/- 2.1	0.69
Abdominal Circumference (cm)	100.7	+/- 4.6	104.9	+/- 6.3	0.59

Data represents mean +/- standard error.

### Statistics

All statistical tests were performed using the R (version 3.0.2,[21]). To correct for multiple hypotheses, p-values were adjusted by the method of Benjamini and Hochberg [22] and referred to in this manuscript as q-values. The age adjusted, p-value corrected for multiple is denoted as q. Statistical significance was set at p/q<0.05 for most comparisons except for GSEA analysis in which a p<0.25 was used.

Descriptive statistics such as means and standard deviations were determined for clinical measurements. Student’s t-test was used to test the difference in means of these measurements between control and acromegaly patients. Normality assumption was checked by Shapiro-Wilk test. Wilcoxon rank sum test was performed for HOMA-IR score, insulin levels and the 14:0, 16:0, 20:0 ceramides and the C16:0 glucosylceramide species as these data were not normally distributed. Welch’s t-test was used for basal lipolysis since the equal variance assumption was rejected by Levene's test (car package version 2.0–19 [23]). Correlation coefficients were calculated by Pearson's product-moment.

## Results and Discussion

### Patient Characteristics

Clinical and metabolic measurements were obtained for 9 acromegaly patients and 11 controls. Patient characteristics are shown in Table 1. There was no statistically significant difference in body mass index (BMI), abdominal circumference or weight. Acromegaly patients were younger (p = 0.011) and taller than their controls (p = 0.036).

### Acromegaly Patients Were More Insulin Resistant and Had Higher Lipolysis

**Fig 1:**
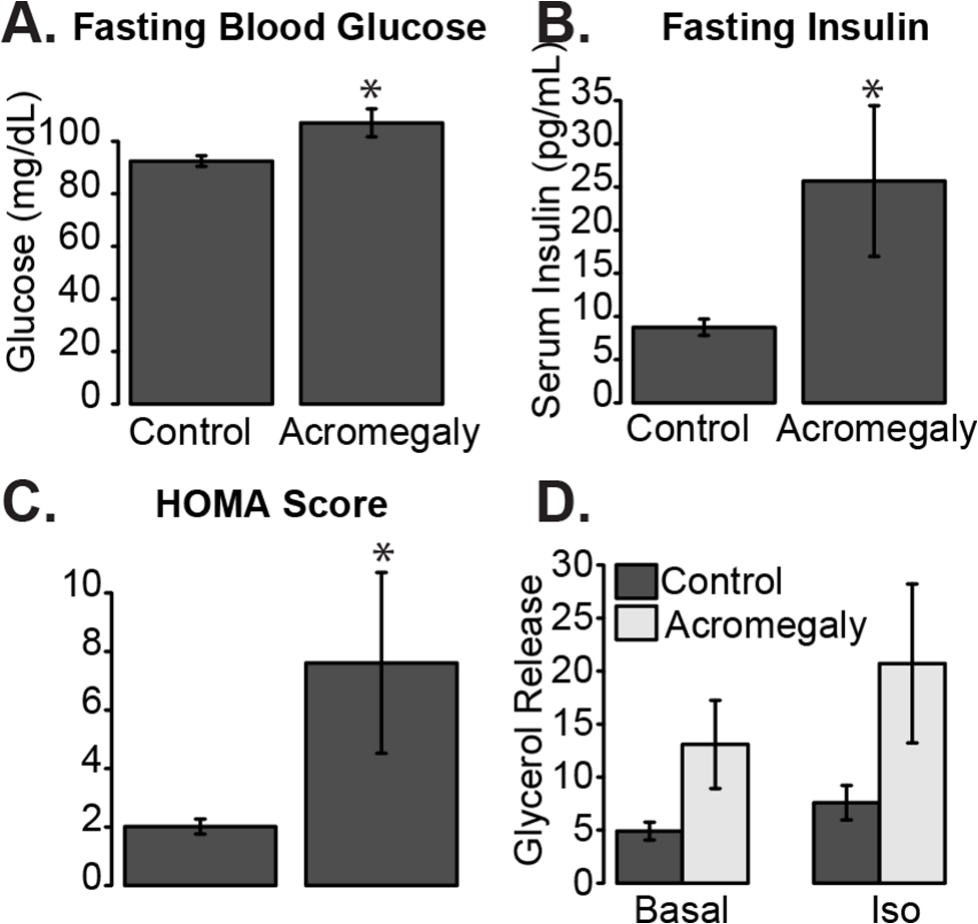
Acromegalic patients have reduced insulin sensitivity and trend for higher lipolytic activity than their controls. A) Fasting blood glucose levels. B) Fasting insulin levels. C) HOMA-IR score from Control or Acromegaly subjects. D) *ex vivo* lipolysis as measured by glycerol release from excised white adipose tissue from control or acromegaly patients left untreated (Basal) or after stimulation with 30 nM isoproterenol (Iso). Data is presented as mean +/- standard error of the mean. Asterisk indicates q<0.05.

Acromegaly patients had elevated fasting glucose levels (p = 0.013) and higher fasted insulin (p = 0.012, Fig 1A and 1B). When combined, we observed higher HOMA-IR scores in the acromegalic patients than in the controls (p = 0.001, Fig 1C), reflecting a significant decrease in insulin sensitivity in the acromegaly patients, consistent with previous clinical findings [3].

Subcutaneous adipose tissue chunks for lipolysis assay were available from 6 acromegaly patients and 9 controls. The results suggested that acromegaly patients may have higher basal lipolysis (p = 0.11), and higher lipolysis in the presence of isoproterenol (p = 0.058) even though they did not achieve statistical significance (Fig 1D). These data are consistent with previous reports linking GH signaling with increased lipolysis [24].

### Transcriptomic Analysis

To determine which genes are altered in adipose tissue in acromegaly subjects, we performed a transcriptomic analysis of subcutaneous adipose tissue mRNA from 7 acromegalic patients and 11 controls. Patients separated along the first principal component approximately based on their age, with 10/11 controls in one group and 7/8 of the acromegalic patients on the other group (S1 Fig). This suggests that the major molecular differences between these groups can be explained by the presence or absence of acromegaly.

**Fig 2:**
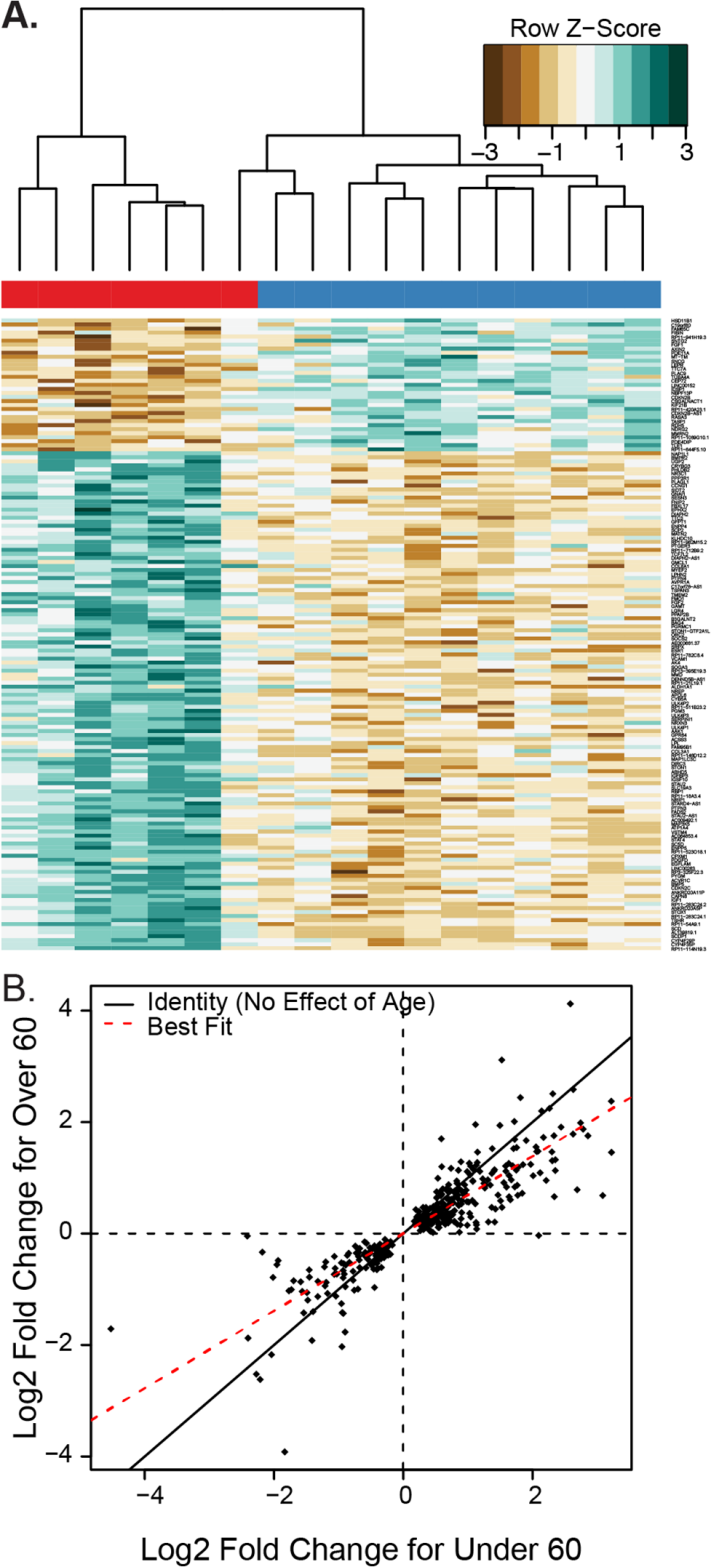
Differential expression of genes in white adipose tissue from subjects with acromegaly compared to controls. A) Heatmap of the differentially expressed genes in white adipose tissue. Individual values are colored as the log fold change for a particular gene in a particular subject compared to the average expression of that gene across all cohorts, with brown indicating less expression and green indicating more expression (designated in the key as Row Z-score). The bar across the top indicates the subject’s diagnosis, red for acromegaly and blue for controls. B) Scatterplot showing the log2 fold change for genes which had a statistically significant difference (q<0.05) between acromegaly and control subjects. Each dot represents the log2 fold change for acromegaly for a gene in the under 60 and 60 or over cohorts. The solid line represents a slope of 1, which would imply no difference in fold change between age groups. The red line is a best fit line with a lower slope, showing that on average the fold change for older patients is smaller than the fold change for the under-60 patients.

After correcting for age, we identified 418 genes that had significantly different expression in acromegaly. Of these, 198 genes were down-regulated and 290 were up-regulated in adipose tissue from the acromegalic patients. These transcripts form a signature identifying transcriptional differences in adipose tissue in response to long-term exposure to GH or indirectly to IGF-1 (Fig 2A and S1 and S2 Tables).

In general, gene expression changes in acromegalic patients over 60 were smaller than in patients under 60 (Fig 2B). Among genes that had significantly different expression, the fold change for a gene was 25 +/- 2.3% higher in the younger cohort than the older cohort. This effect was statistically significant via a Wilcoxon-Rank Sum test (p = 1.4 x 10^−11^).

Gene set enrichment analysis testing KEGG pathways [25,26] showed enrichment of genes in the categories involved in metabolism, including upregulation of genes involved in the TCA cycle, fatty acid metabolism, biosynthesis of unsaturated fatty acids as well as genes which regulate the cell cycle. We also observed downregulation of genes involved in pathways of GPCR signaling, MAPK signaling, inflammation and protein synthesis (S3 Table). We also examined the transcription factor networks that may underlie these changes in mRNA levels. We identified an up-regulation of several candidate transcription factors and microRNA’s (S4 Table) whose targets are significantly altered in acromegalic subcutanous white adipose tissue after adjusting for age. Notably among these are an up-regulation of E2F, GATA-1, MEF-2 and CREB targets and a down-regulation of AP1, STAT1, 3, 4 and 6, PPARα, NF-κB and SRF responsive genes.

### Established GH Responsive Genes Are Up-Regulated in Adipose Tissue from Acromegaly Patients

**Fig 3:**
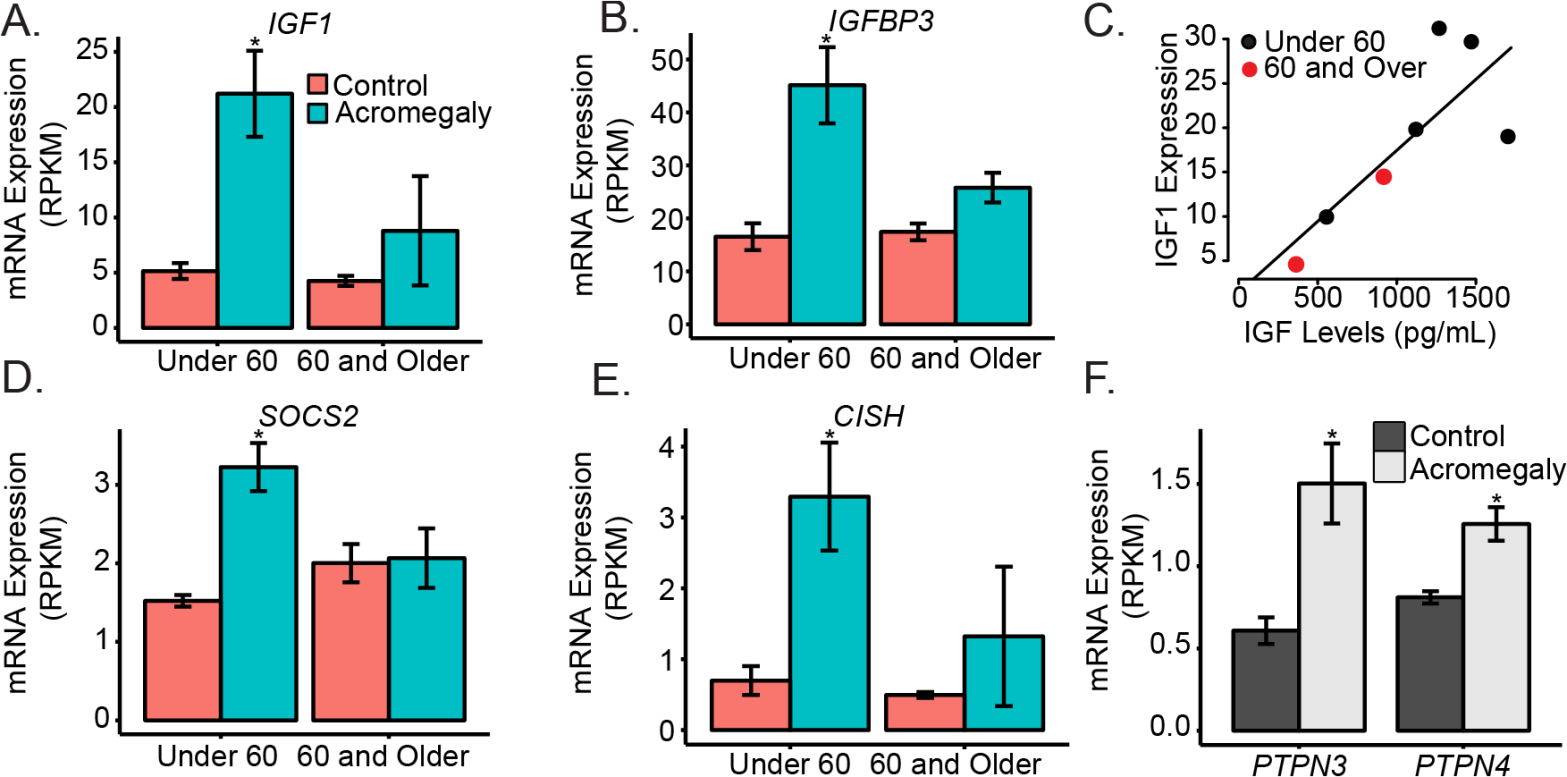
GH targets are differentially expressed in acromegaly subjects. A) mRNA Expression of A) *IGF1* and B) *IGFBP3* transcript levels in adipose tissue from control and acromegalic patients (C) Comparason between *IGF1* mRNA and IGF-1 serum levels in patients with acromegaly (D and E) Expression of mRNA for suppressors of growth hormone signaling (F) and Expression of tyrosine phosphatases associated with growth hormone signaling. Asterisks indicate q<0.05 for the separated under 60 and 60 or over cohorts for panels A, B, D and E and for the age adjusted combined analysis for panel F. Barplots are presented as mean +/- standard error of the mean.

Since acromegaly is caused by an overproduction of GH, we first analyzed known GH responsive genes. We found that expression of previously reported GH responsive genes, including *IGF1* (3.5 fold q = 1.65 x 10^−6^), and *IGFBP3* (2.3 fold, q = 0.0002) are elevated in acromegalic patients (Fig 3A and 3B). IGF-1 has been shown to be induced in adipocytes exposed to GH [27], while there were no previous reports regarding *IGFBP3* induction in adipose tissue. The confirmation of these previously reported acromegaly or GH-induced transcriptional changes strengthens our interpretation of other transcriptional changes. Neither the growth hormone receptor (GHR) nor the IGF-1 receptors (*IGF1R*, *IGF2R)* was significantly altered in acromegalic adipose tissue.

There was a correlation between *IGF1* mRNA and levels of IGF-1 in serum in the acromegaly patients (R^2^ = 0.51, p = 0.043; Fig 3C), reflecting that increased induction of *IGF1* in adipose tissue is similar in its extent to serum IGF1 induction. Serum IGF1 is primarily thought to be derived from liver tissue due to the observation that serum IGF-1 levels are reduced 75% in a liver specific IGF-1 knockout [28]. Our data demonstrates that expression of the adipose tissue *IGF1* gene correlates well with that of serum IGF-1. Note that the older subjects had lower serum IGF-1 than the younger subjects, indicating that circulating IGF-1 levels may correlate with generally reduced transcriptional changes observed in the older acromegalic patients (Fig 3C).

### A Novel Negative Feedback Loop Is Induced by Chronic Exposure to High GH/IGF-1 Levels

*SOCS2* AND *CISH*, both suppressors of cytokine signaling that have been shown to be important in down-regulating GH signaling, are up-regulated in acromegaly (1.7 and 2.3 fold respectively (q = 0.003 and q = 0.00014, Fig 3D and 3E). These data suggest that feedback mechanisms may be more active in younger patients, potentially either due to improved flexibility or reduced duration of the disease. These have been shown to be induced in liver and muscle by GH [29], and *SOCS2* has also been reported to be induced in adipocytes by GH [27,30]. We observed no significant differences in any PIAS genes.

We observed induction of the tyrosine phosphatases, *PTPN3* (also called PTP-H1, 2.5 fold higher q = 0.0028), *PTPN4* (1.6 fold q = 0.00014) and *PTPN13* (1.3 fold, q = 0.038) in acromegaly (Fig 3F). *PTPN3* has been reported to bind GH receptor *in vitro* in the presence of GH [31], and its overexpression reduces STAT5 signaling in response to GH [32]. *Ptpn3* Knockout mice have excessive GH activity, as demonstrated by excessive growth accompanied by a strong induction of liver *IGF1* mRNA and serum IGF-1 [32]. This is the first report of enhanced abundance of PTPN3 mRNA in response to GH/IGF-1 exposure. The increased expression of *PTPN3* that we have observed in acromegaly suggests that this may be an additional negative feedback pathway induced by GH and reducing GH/IGF-1 signaling.

### Genes Controlling DNA Replication, Proliferation and Apoptosis

We observed a difference in expression of several different genes regulating cellular proliferation in acromegalic subjects. The KEGG category containing DNA replication was enriched in acromegalic white adipose tissue, (S3 Table). Expression of Cyclin C (*CCNC;* 1.2 fold q = 0.022), and Cyclin E (*CCNE1;* 2.9 fold, q = 6.5 x 10^−5^) which are important for transition from G1 to S, were increased in acromegalic patients, and the negative regulator, cyclin dependent kinase inhibitor B (*CDKN2B*) was decreased (40% reduced fold q = 0.016, S2A Fig). *CDKN2B* has also been identified as a diabetes susceptibility gene identified in GWA studies [33,34].

Additional DNA replication genes that were induced were nucleosome assembly protein 1-like 1 (*NAP1L1*, 1.3 fold q = 0.025) and origin recognition complex, subunit 2 (*ORC2*, 1.7 fold q = 0.0044), which are important for DNA replication, and the anti-apoptotic regulators *BAG4* (BCL2-associated athanogene 4. 1.7 fold q<10^−4^) and *CAPN6* (calpain 6, 3.7 fold q = 0.0011) were also induced (S2 Fig). Together these implicate increased cell division, potentially of immune, vascular or pre-adipocyte cells in adipose tissue depots.

Apoptosis signal-regulating kinase 1 (*MAP3K5*) expression is higher in acromegaly (2.8 fold q<0.0004), and there is also higher expression of its downstream substrates p38 α (*MAPK14* 1.2 fold q = 0.012), p38*δ* (*MAPK13*, 2.7 fold q<10^−4^). The effect of GH or IGF-1 on these transcripts has not been reported before, and they could account for the effects of enhanced cell proliferation and apoptosis in response to GH/IGF-1 [35].

### Transcriptional Changes Regulating Lipid Metabolism and Localization that May Contribute to Enhanced Lipolysis

**Fig 4:**
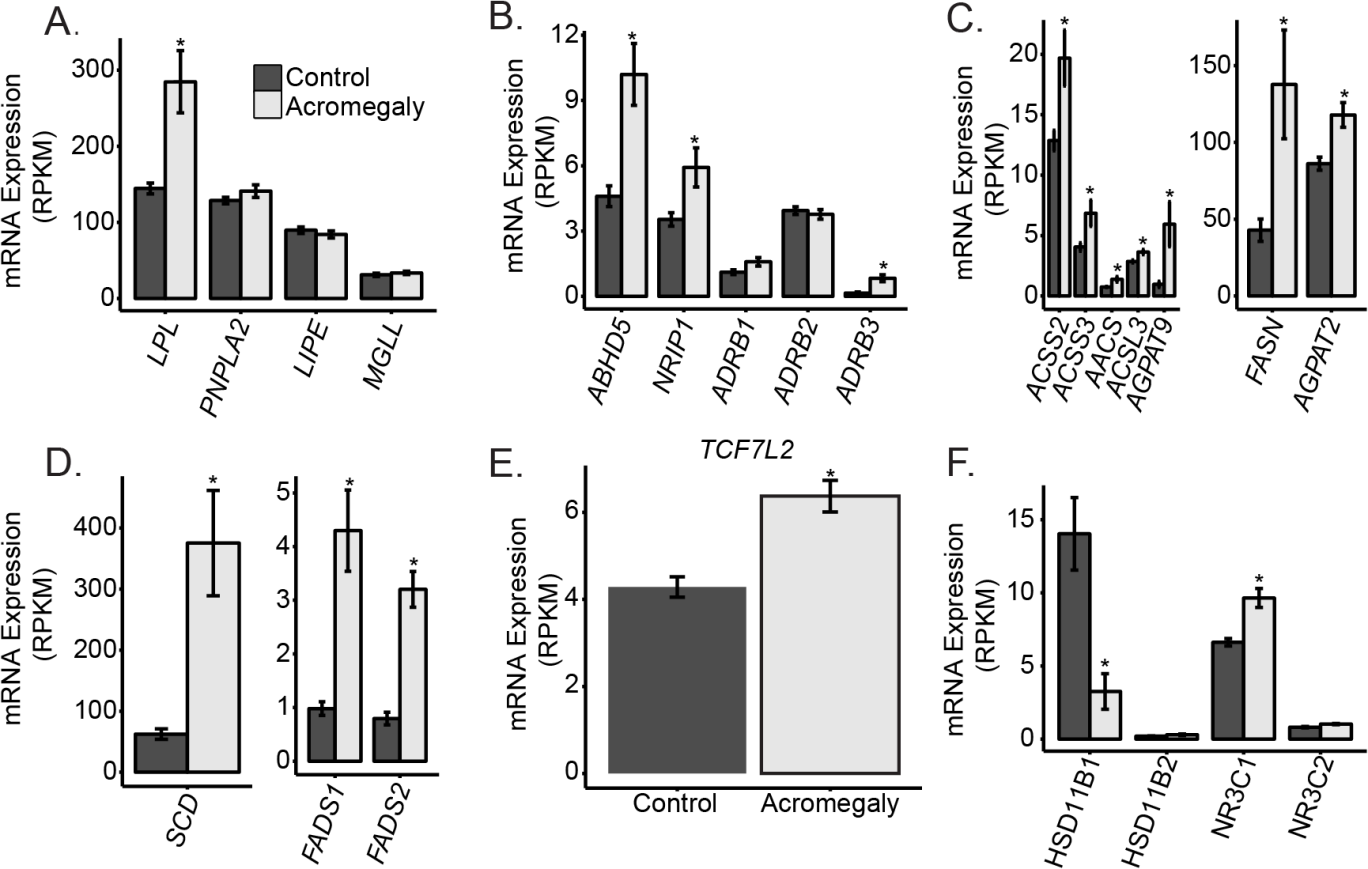
Expression changes of selected genes, potentially responsible for lipolytic or insulin sensitivity alterations in acromegaly patients. mRNA Expression profile of genes potentially involved in lipid catabolism (A), regulation of lipolysis (B), fatty acid synthesis (C), fatty acid desaturation (D), *TCF7L2* (E), and glucocorticoid signaling (F). Asterisks indicate q<0.05. Data indicates mean +/- standard error of the mean.

To determine the potential causes of the increased lipolysis observed in Fig 1D, we examined the expression of human lipases in these adipose tissues. We observed no significant difference in expression of the three classical triglyceride lipolysis enzymes hormone sensitive lipase (*LIPE*), adipose triglyceride lipase (*PNPLA*) or monoglycerol lipase (*MGLL*; Fig 4A). Lipoprotein lipase (*LPL*), the lipase important for lipolysis of triglycerides in apolipoproteins, was significantly more highly expressed in acromegaly patients (2.0 fold, q = 0.004). A strong induction of LPL expression in response to GH and absence of change in HSL were demonstrated before in a preadipocyte cell line [36–38] and in adipose tissue biopsies from GH deficient patients after treatment with GH [11]. Studies that addressed LPL enzymatic activity and not expression have found either no change or even a reduction in LPL activity in response to GH treatment of human adipocytes *in vitro* [39,40].

Although neither Hormone Sensitive Lipase (*LIPE*) nor ATGL (*PNPLA2*) were altered, two direct regulators of HSL and ATGL activity in adipocytes, abhydrolase domain containing 5 (*ABHD5*, also called CGI58 [41]), and nuclear receptor interacting protein 1 (*NRIP* (also called RIP140 [42]) were expressed at higher levels in adipose tissue from acromegaly patients (2.3 fold q = 0.0016 and 1.7 fold, q = 0.043 Fig 4B). CGI58 is an allosteric activator of lipolytic activity and RIP140 regulates CGI58’s activity, therefore these data suggest that their transcriptional up-regulation could contribute to the induction of lipolysis by GH/IGF-1. NRIP1 has also been proposed to be a transcription regulator of genes involved in lipid and glucose metabolism [42] and its induction could contribute to additional metabolic effects of GH/IGF-1 including disrupted glucose metabolism. We also examined *CIDEA/B/C* and *G0S2*, which have also been proposed to be positive regulators of lipolysis [43]. While there were no changes in the former, we did observe a non-significant elevation in *GOS2* (1.53 fold, q = 0.246; S2C Fig).

We next examined the expression of G-protein coupled receptors that induce lipolysis. The β3 adrenergic (*ADRB3*) was more highly expressed in acromegaly patients compared to the controls (5.2 fold, q = 0.0064). The β1 receptor was also more highly expressed in acromegalic adipose tissue though it did not reach statistical significance (1.5 fold, q = 0.20; Fig 4B) suggesting a potential sensitization of these patients to adrenergic stimuli may underlie the enhanced *ex vivo* lipolysis.

In contrast to the lipolytic phenotype of acromegalic patients, several fatty acid and triglyceride synthesis genes were expressed at higher levels in acromegaly patients (Fig 4C). These include *ACSS2* (1.6 fold, q = 0.044) and *ACSS3* (1.7 fold, q = 0.064), which catalyze the activation of acetate for use in lipid synthesis. We also observed elevations in Acetoacetyl Co-A synthase (*AACS;* 1.9 fold, q = 0.0066), Acetyl Co-A carboxylase (*ACACA*, 1.7 fold q = 0.039), and Acyl-CoA synthetase long-chain family member 3 *ACSL3* (1.3 fold, q = 0.045), which convert long-chain fatty acids into fatty acyl-CoA esters. In addition, we also observed an elevation in fatty acid synthase *FASN* (1.7 fold, q = 0.01) in the acromegaly patients. The first two steps in triglyceride synthesis from fatty acids are catalyzed by glycerol-3-phosphate acyltransferase 9 (*AGPAT1*,*2*,*3* and *5*) and 1-acyl-sn-glycerol-3-phosphate acyltransferase (*AGPAT9*). We observed transcriptional up-regulation of both *AGPAT2* (42% increased, q = 0.02) and *AGPAT9* (7.6 fold increased, q = 2.7 x 10^−4^) in acromegaly patients. Via pathway analysis, both fatty acid metabolism and unsaturated fatty acid biosynthetic pathways were up-regulated in the acromegaly patients (see S2 Table and below). This up-regulation may represent compensation by the adipose tissue due to enhanced lipid breakdown and oxidation in this and other tissues in acromegaly patients.

With respect to glycogen metabolism, expression of muscle glycogen phosphorylase (*PYGM*) was 3.2 fold higher (q = 0.00078) in the acromegaly patients (S2D Fig). The significance of glycogen in adipose tissue, or changes in glycogen content in acromegalic adipose tissue have not been characterized, but these findings would predict that the rates of glycogenolysis in acromegalic white adipose tissue would be elevated.

### Transcripts Altered in Acromegaly that May Contribute to Insulin Resistance

One of the most pronounced differences between the acromegaly patients and the controls was a greater than 4 fold higher expression (q<1x10^-5^; Fig 4D) of the three fatty acid desaturases—stearoyl-CoA desaturase (*SCD*, delta-9-desaturase), fatty acid desturase 1 (*FADS1*, delta-5-desaturase) and fatty acid desturase 2 (*FADS2*, delta-6-desaturase). *SCD* products and *FADS2* mRNA have also recently been shown to be induced by GH in mice [44]. The change in expression of these enzymes could be a possible link between acromegaly and insulin resistance due to an elevation of unsaturated fatty acids, as activity of *FADS1* and *FADS2* are associated with metabolic syndrome [45,46].

We observed no decrease in expression of canonical transcripts important for insulin signaling and response to insulin in adipocytes, including insulin receptor (*INSR*), *IRS1*, *IRS2*, *AKT1-3*, or GLUT4 (*SLC2A4*; see S3A Fig). This indicates that the observed insulin resistance is not caused by mRNA decreases in these genes. In fact, the KEGG category containing insulin signaling genes was generally up-regulated in these tissues (S3 Table). *AKT1* was significantly higher (1.3 fold, q = 0.006) and the remainder of these genes trended to be more highly expressed in the adipose tissue from the acromegalic and insulin resistant patients, potentially underlying a transcriptional up-regulation that compensates for an alternative insulin resistance mechanism.

One previously identified candidate is the phosphoinositide-3-kinase, regulatory subunit 1 (*PIK3R1*, also called p85α), which was induced by GH in mouse adipose tissue and thought to contribute to GH-induced insulin resistance [47]. In our study *PIK3R1* expression was not significantly different in the acromegaly patients, though it was modestly increased (25% increased, q = 0.23).

The ERK kinase pathway was down-regulated in the acromegaly patients including a 2–4 fold lower expression of the downstream transcription factors *FOS*, *JUN*, *JUNB* (q<0.04, S2 Table and S3B Fig). Jun and Fos form the transcription factor AP1, which drives transcription of many targets involved in differentiation, proliferation and apoptosis [48]. Globally, we also found that AP1 targets were down-regulated. (ie, NES = -3.30, q<1 x 10^−4^ for V$AP1_Q4_01, S4 Table).

The cytokine modulators *STAT6* and the pro-inflammatory protein kinase IKKβ (*IKBKB*) are expressed at lower levels (~18% reduced, q = 0.0034 for *STAT6* and q = 0.009 for *IKBKB*). Furthermore, the pro-inflammatory cytokines *IL1B*, *IL6* and *CCL2* (MCP-1) and the pro-inflammatory protein kinase *IKBKE* all trend towards lower expression, and genes from several KEGG pathways involved in inflammation were expressed at lower levels in the acromegaly cohort (S2 Table and S3 Table and S3C Fig). These data support the hypothesis that insulin resistance in these patients is not due to enhanced inflammatory signaling.

To test biochemically whether ceramides may play a role in the acromegaly associated insulin resistance, we took a lipidomics approach to analyze ceramide and glucosylceramide species from the adipose tissue explants of these patients. Elevated ceramides have been proposed to mediate insulin resistance by several models [49–52]. We observed a modest down-regulation of the mRNA levels of glycosylsphingolipid metabolic genes in our data (normalized enrichment score = -0.86 q = 0.71). We then measured ceramide species from 7 acromegaly patients and 11 control patients directly and observed no statistically significant changes in any ceramide species (S4 Fig, q-values all >0.25). We did however, detect modest elevations of C16:0, C18:0 and C24:0 ceramide species in subcutanouse adipose tissue from acromegalic patients. We therefore do not have strong evidence to support the hypothesis that ceramide elevations are causative of insulin resistance in acromegalic white adipose tissue.

*TCF7L2*, a transcription factor regulating many metabolism genes that is also a diabetes susceptibility gene [53] is up-regulated in the acromegaly patients (1.5 fold, q = 0.00045, Fig 4E). Mice with liver specific knockout of *Tcf7l2* are hypoglycemic, while transgenic mice overexpressing liver *Tcf7l2* are hyperglycemic [54]. *TCF7L2* in subcutaneous fat is higher and expression of splice isoforms is reduced in subcutaneous fat and in liver following bariatric surgery [55]. Higher expression of TCF7L2 could also therefore be linked to insulin resistance in acromegaly.

### Glucocorticoid Regulation

11βHydroxysteroid dehydrogenase 1 (*HSD11B1*), the enzyme that activates transformation of cortisone to cortisol, was reduced over 4 fold in acromegaly patients (q = 0.0048, Fig 4F). The down-regulation of expression and activity of this enzyme by GH/IGF1 has been confirmed *in vitro* [56], in GH deficient patients treated with GH [57] and in acromegaly patients [58,59]. In addition, we found higher expression of the glucocorticoid receptor (*NR3C1*, 1.5 fold q = 0.00013) in acromegaly patients (Fig 2F). Glucocorticoid receptor expression is repressed by cortisol [60], so the higher expression may be due to the reduced local cortisol levels caused by lowered *HSD11B1*.

## Summary

In this study we have described a transcriptional signature in adipose tissue from subjects with acromegaly. We identified 418 adipose tissue genes altered in acromegaly patients. Some of these genes may be direct targets of increased GH or IGF-1 signaling in adipose tissue, whereas others may be secondary adaptations to this condition.

Interestingly, we observed more modest gene expression changes in general for older acromegalic patients than for younger patients. We are unable to determine from our study how long patients were acromegalic prior to our study, so one possibility is that the older patients have had longer to adapt to elevated GH levels. Alternatively, elevated GH/IGF-1 signaling may play a stronger role in younger patients. It should be noted, however that this exploratory finding was limited since we only had 2 acromegalic patients over 60 in our study, so these age-dependent findings will need to be reproduced in a larger cohort.

The fact that the patients consistently had a relatively uniform change of expression of these genes suggests that we are able to draw valid conclusions about adipose tissue in acromegalic patients even from this small cohort. Furthermore, as mentioned throughout, our data agree with several previous studies in animal models and patients. The confirmation of these previously reported GH-dependent transcriptional changes strengthens our interpretation of other transcriptional changes. One potential caveat to our approach is the use of patients with a non-secreting adenoma as the control group. To avoid the possible effects of hypopituitarism on adipose tissue we excluded patients with pituitary hormone deficiencies. We chose to include this as the control group as these samples not only collected in an identical manner from the same surgeons and processed identically, but also controls for potential non-secreting effects of pituitary tumor growths in the acromegaly subjects.

A potential caveat is the potential confounding effect of anti-diabetic or anti-growth hormone medications. Only one acromegalic patient was on somatostatin, and his IGF-1 levels were non-responsive. Our exclusion criteria included any glucocorticoid treatment. One patient in each group was on metformin as an antidiabetic medication, so we do not feel that this affected our overall conclusions. Another potential limitation was our inability to reanalyze the samples by a secondary method for gene expression or to validate our findings at the protein level, due to a lack of sample. We could not analyze a second cohort due to the rarity of this disease.

These data provide a variety of novel transcriptional changes that may be causative of the co-morbidities associated with acromegaly. Further studies in animals and cells using knockout or overexpression of specific transcripts may verify which of the changes is crucial in metabolic effects of GH in adipose tissue.

## Supporting Information

S1 Fig Principal component analysis of normalized transcript levels. Normalized counts were transformed via a regularized log transformation then principal components were calculated. Samples were then colored based on age and diagnosis. The dotted line indicates the grouping of samples into groups based on their disease state.
(TIF)

S2 Fig Expression changes of selected other transcripts. mRNA Expression profile of differentially expressed genes involved in A) MAPK Signaling, B) Cell cycle control, C) Lipolysis and D) Glycogen metabolism. Asterisks indicate q<0.05. Barplots are presented as mean +/- standard error of the mean. mRNA Expression is in units of RPKM (reads per kilobase per million reads).
(TIF)

S3 Fig Expression changes of selected insulin signal transduction genes. mRNA Expression profile of genes involved in insulin signaling (A) and inflammation (B). Asterisks indicate q<0.05. Barplots are presented as mean +/- standard error of the mean. mRNA Expression is in units of RPKM (reads per kilobase per million reads).
(TIF)

S4 Fig Ceramide levels in adipose tissue from control and acronegalic patients. Ceramide (Cer) or glucosylceramide levels (GluCer) were determined as described in the methods and materials section. The number indicates the fatty acid species associated with each lipid. All values are normalized such that control values are equal to 1. Data indicates mean +/- standard error of the mean.
(TIF)

S1 Table Expression changes between control and acromegaly subjects. Calculated expression, and expression changes for each gene are shown along with raw p-value, adjusted p-values and the fold change.
(CSV)

S2 Table Age adjusted gene expression changes between control and acromegaly subjects. Patients were grouped into under-60 and 60 and over as the age and genes were first analyzed with age group as a covariate then after that adjustment, the disease state was taken into account. Three log2 fold changes and p-values are presented. The age adjusted effect of Acromegaly (AcrovsControl), the effects if acromegaly in only the under 60 group (AcrovsCon_0.60) and the effects of acromegaly in only the above 60 group (AcrovsCon_60.100).
(XLS)

S3 Table Gene set enrichment analysis of GO and KEGG pathways. Size is the total size of the KEGG category, NES is the normalized enrichment score, NOM p-value is the raw p-value and FDR q-value is corrected for multiple observations. Gene details lists the specific genes which led to the enrichment of this category in our data. A negative enrichment score indicates down-regulation of the category in acromegaly.
(XLS)

S4 Table Gene set enrichment analysis of transcription factor and miRNA pathways. These categories indicate that target genes regulated by these factors are altered in acromegalic white adipose tissue. Size is the total size of the category, NES is the normalized enrichment score, NOM p-value is the raw p-value and FDR q-value is corrected for multiple observations. Gene details lists the specific genes which led to the enrichment of this category in our data. A negative enrichment score indicates down-regulation of the category in acromegaly.
(XLS)
