# Supplementary figures and images for: Gene Expression Signature in Adipose Tissue of Acromegaly Patients
Source: PLoS One. 2015 Jun 18;10(6):e0129359. doi: 10.1371/journal.pone.0129359 (PMC4472931; doi:10.1371/journal.pone.0129359)

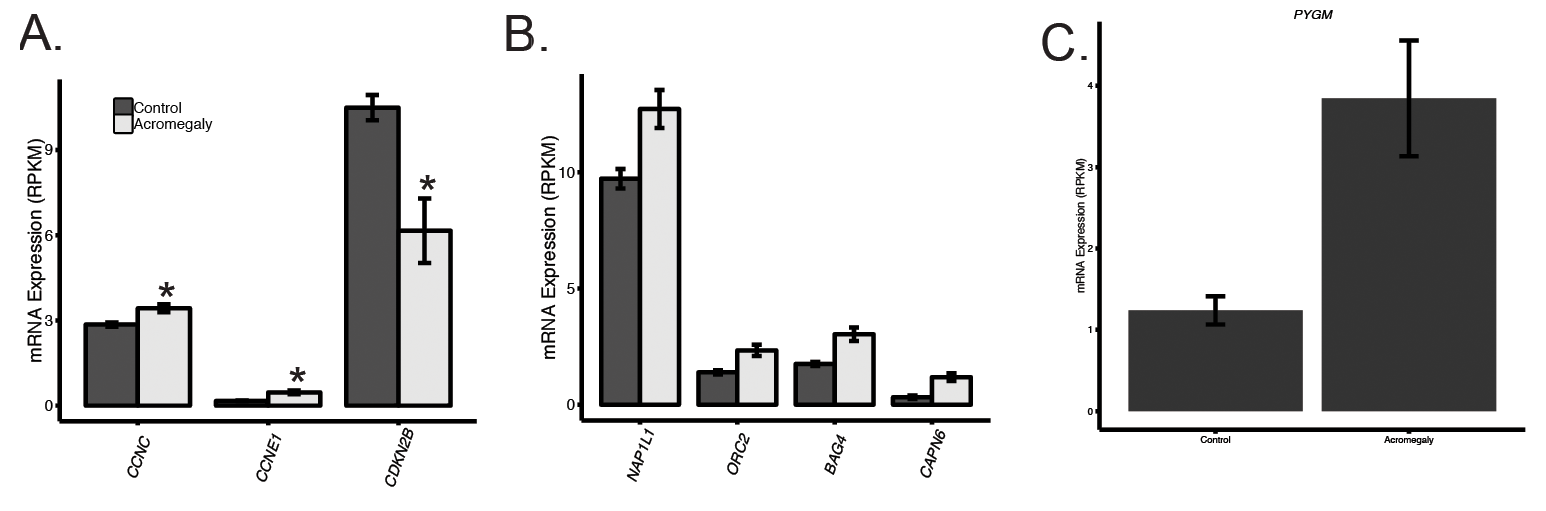

Supplement: S1 Fig — Normalized counts were transformed via a regularized log transformation then principal components were calculated. Samples were then colored based on age and diagnosis. The dotted line indicates the grouping of samples into groups based on their disease state. (TIF) [file pone.0129359.s001.tif]

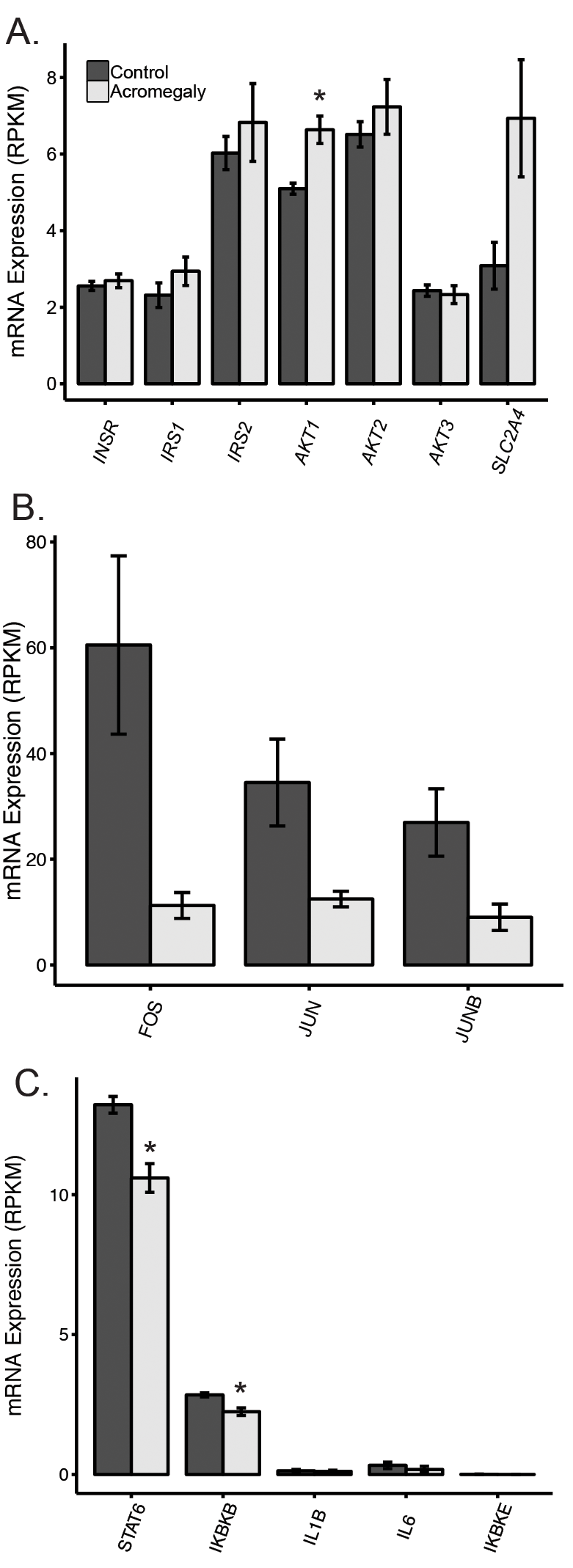

Supplement: S2 Fig — mRNA Expression profile of differentially expressed genes involved in A) MAPK Signaling, B) Cell cycle control, C) Lipolysis and D) Glycogen metabolism. Asterisks indicate q<0.05. Barplots are presented as mean +/- standard error of the mean. mRNA Expression is in units of RPKM (reads per kilobase per million reads). (TIF) [file pone.0129359.s002.tif]

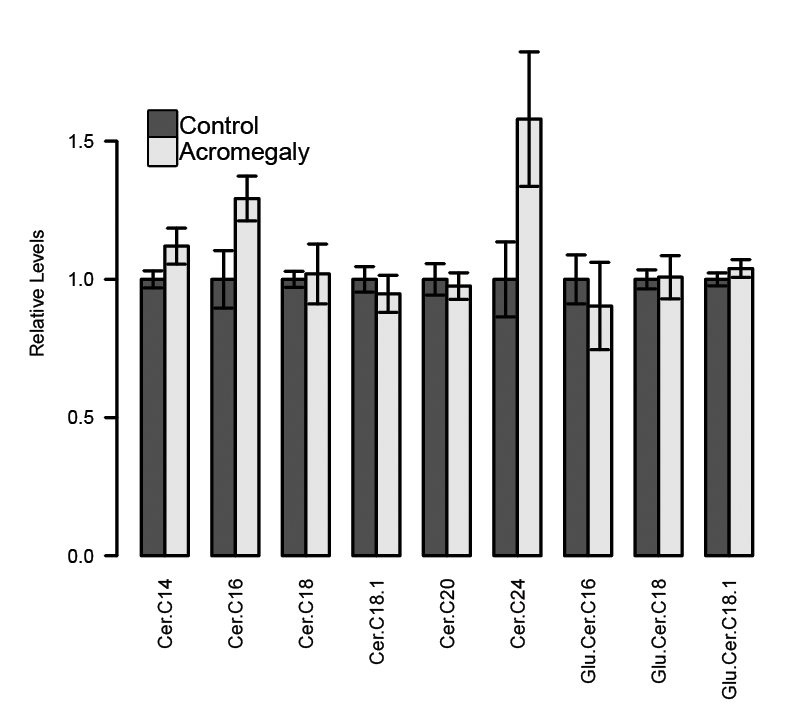

Supplement: S3 Fig — mRNA Expression profile of genes involved in insulin signaling (A) and inflammation (B). Asterisks indicate q<0.05. Barplots are presented as mean +/- standard error of the mean. mRNA Expression is in units of RPKM (reads per kilobase per million reads). (TIF) [file pone.0129359.s003.tif]

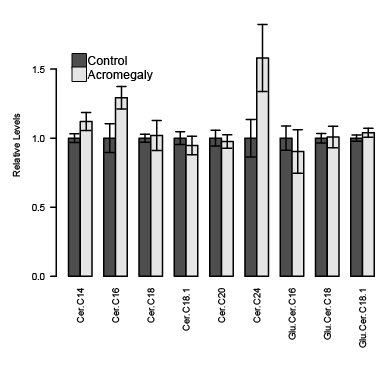

Supplement: S4 Fig — The number indicates the fatty acid species associated with each lipid. All values are normalized such that control values are equal to 1. Data indicates mean +/- standard error of the mean. (TIF) [file pone.0129359.s004.tif]
